# Using Raman spectroscopy and chemometrics to identify the growth phase of *Lactobacillus casei* Zhang during batch culture at the single-cell level

**DOI:** 10.1186/s12934-017-0849-8

**Published:** 2017-12-23

**Authors:** Yan Ren, Yuetong Ji, Lin Teng, Heping Zhang

**Affiliations:** 1 Key Laboratory of Dairy Biotechnology and Engineering, Education Ministry of P. R. China, Department of Food Science and Engineering, Inner Mongolia Agricultural University, Hohhot, 010018 People’s Republic of China; 2 Key Laboratory of Dairy Products Processing, Ministry of Agriculture, Inner Mongolia Agricultural University, Hohhot, 010018 People’s Republic of China; 3 Single-cell Center, CAS Key Laboratory of Biofuels, and Shandong Key Laboratory of Energy Genetics, Qingdao Institute of Bioenergy and Bioprocess Technology, Chinese Academy of Sciences, Qingdao, 266101 Shandong People’s Republic of China

**Keywords:** *Lactobacillus casei* Zhang, Growth phases, Single-cell Raman spectrometry, Chemometrics

## Abstract

**Background:**

As microbial cultures are comprised of heterogeneous cells that differ according to their size and intracellular concentrations of DNA, proteins, and other constituents, the detailed identification and discrimination of the growth phases of bacterial populations in batch culture is challenging. Cell analysis is indispensable for quality control and cell enrichment.

**Methods:**

In this paper, we report the results of our investigation on the use of single-cell Raman spectrometry (SCRS) for real-time analysis and prediction of cells in different growth phases during batch culture of *Lactobacillus (L.) casei* Zhang. A targeted analysis of defined cell growth phases at the level of the single cell, including lag phase, log phase, and stationary phase, was facilitated by SCRS.

**Results:**

Spectral shifts were identified in different states of cell growth that reflect biochemical changes specific to each cell growth phase. Raman peaks associated with DNA and RNA displayed a decrease in intensity over time, whereas protein-specific and lipid-specific Raman vibrations increased at different rates. Furthermore, a supervised classification model (Random Forest) was used to specify the lag phase, log phase, and stationary phase of cells based on SCRS, and a mean sensitivity of 90.7% and mean specificity of 90.8% were achieved. In addition, the correct cell type was predicted at an accuracy of approximately 91.2%.

**Conclusions:**

To conclude, Raman spectroscopy allows label-free, continuous monitoring of cell growth, which may facilitate more accurate estimates of the growth states of lactic acid bacterial populations during fermented batch culture in industry.

## Background

Cell heterogeneity resulting from environmental pressure implies the co-existence of cells at different physiological states [1, 2]. Being able to characterise and predict the physiological state of individual cells in a microbial population is of great importance in a biotechnological fermentation because (1) the physiological state of the individual cell is the only factor that determines the yield of any product, provided that the required nutrients are present in non-limiting amounts, and (2) the knowledge of the physiological state is a prerequisite for tuning fermentation for optimal performance [3].

This knowledge has traditionally been acquired indirectly, by measuring parameters such as pH, cell density, sugar utilisation and product formation. However, as techniques in molecular biology have improved considerably, the physiological state of cells during the fermentation process has been addressed in much greater detail, which can provide a more accurate and descriptive representation of the population than average values attained from traditional techniques [4]. Microscopy and flow cytometry have advanced substantially in recent decades, and are now essential tools for monitoring the physiological heterogeneity of microbial populations at the single-cell level. However, both methods rely on fluorescence monitoring for measuring cellular parameters, such as reporter systems where the cellular component of interest is fluorescent (e.g. reporter proteins such as green fluorescent protein). In addition, these methods also allow the monitoring of other intrinsic cell properties (e.g. cell size,) or structural/functional parameters (e.g. membrane integrity, and DNA content), by using different staining procedures [3]. Various spectroscopic methods have also been applied to monitor microbial populations. Regarding single-cell analysis, Raman spectroscopy holds promise due to its non-destructive nature, and the ability to provide information at the molecular level without the use of stains or radioactive labels [5].

Raman spectroscopy is an optical, marker-free technology that allows continuous analysis of dynamic growth events in single cells by investigating the overall molecular constitution of individual cells within their physiological environment. Interestingly, this technology is not dependent on defined cellular markers, and it can be adapted for heterogeneous cell populations [6]. In Raman spectroscopy, rare events of inelastic light scattering occur on molecular bonds due to excitation with monochromatic light and generate a “fingerprint” spectrum of the investigated specimen [7, 8]. Although the effect of Raman scattering is weak, the presence of water does not impact Raman spectra, enabling the examination of native biological samples without the need for fixation or embedding procedures and making the technique superior to infrared spectroscopy. For this reason, Raman spectroscopy has been used extensively for a wide variety of applications [9], and it appears to be the most promising spectroscopic method for real-time analysis of complex cell culture systems. Raman spectroscopy has been applied successfully to the monitoring of cell biomass [10]. Additionally, Raman spectroscopy can reveal specific information down to the molecular level, and it offers high potential for the detection and classification of cells of different metabolic states [11–13]. However, no reported studies have applied Raman spectroscopy for real-time monitoring and prediction of metabolic states of lactic acid bacteria (LAB) cells.

In this study, we used the industrial probiotic *L. casei* Zhang as a research object to develop a classification model from the Raman spectra of three different growth phase cells using the Random Forest (RF) method. When trained with 214 spectra originating from three different growth phases, the method showed high mean sensitivity (90.7%) and mean specificity (90.8%) for distinguishing cells of different growth phases of *L. casei* Zhang. Furthermore, more than 91.2% of cells were assigned to the correct cell type, which demonstrates the potential of single-cell Raman spectroscopy (SCRS) for determining the metabolic state of *L. casei* Zhang during fermentation.

## Methods

### Growth and culturing of *Lactobacillus casei* Zhang cells

All chemicals used in this study were obtained from Sigma-Aldrich UK (Dorset, UK). The strain *L. casei* Zhang was isolated from traditional home-made koumiss in Inner Mongolia, China [14]. Cell culturing of *L. casei* Zhang (LABCC 20048) cells was started on an MRS agar plate from − 80 °C stock approximately 48 h prior to the experiment. A single *L. casei* Zhang colony was then inoculated from the plate into 5 mL of MRS broth in a glass tube and incubated at 37 °C in a constant-temperature incubator until the OD_600_ of the cells reached ~ 0.3 (UV/Vis spectrophotometer; GeneQuant 1300). Then, it was re-incubated in another 5 mL of MRS broth in a glass tube and incubated at 37 °C in a constant-temperature incubator. The *L. casei* Zhang growth curve was recorded by sampling the bacterial culture at 0–30-h time steps. The growth curve was constructed from each absorbance measurement at 600-nm wavelength and viable bacteria counting at 2-h intervals.

### Single-cell Raman spectrometry

Cell aliquots were collected just after re-inoculation and from each triplicate group at nine subsequent time points: 0, 2, 4, 8, 10, 14, 18, 24, and 30 h. All cell samples were washed three times with deionised water to remove the culture medium. Cell density was adjusted accordingly to ensure sufficient dispersion of individual cells on the slide. After washing and resuspending, a 1.5-μL cell suspension was transferred onto a calcium fluoride (CaF_2_) slide and air-dried prior to Raman analysis. Raman spectra were obtained using a modified confocal Raman-fluorescent microscope based on the LabRam HR (Horiba Ltd., UK) system, as described in [15]. A 100× magnification dry objective (NA = 0.90, Olympus, UK) was used for sample observation and Raman signal acquisition. A 532-nm Nd:YAG laser (Ventus, Laser Quantum Ltd., UK) was used as the light source for Raman measurement, and the power on the sample was 3–5 mW. The acquisition time for each spectrum was 10 s. Each sample from one time point had three biological replicates. Ten cells were measured by SCRS for each biological replicate of the cell culture. The scattered photons were collected by a Newton EMCCD detector (DU970N-BV, Andor, UK). A 600-groove mm^−1^ diffraction grating was used for most of the measurements (unless otherwise stated).

### Data pre-processing of Raman spectra

Pre-processing of raw Raman spectra, performed with LabSpec 5 software (HORIBA Scientific, Orsay, France) included background subtraction, baseline correction by a second-degree polynomial algorithm, get zero and area normalisation. For each sample, the background spectrum provided for subtraction was generated as the average of five spectra acquired from the slide around the cell. The biochemical fingerprint region (600–1800 cm^−1^) was extracted for the subsequent multivariate analysis to extract the useful information contained in Raman bands from useless noise [16], and a spectral resolution result of ~ 2 cm^−1^ from 592 data points was used for analysis.

### Chemometrics analyses

#### Principle component analysis

The normalised fingerprint regions of SCRS were first subjected to principal component analysis (PCA) for discrimination in Matlab R2010a [17]. For further statistical analysis, the dimension of the dataset was reduced by PCA, and only the first 30 scores were retained. Based on the cosine-distance matrix, analysis of similarities (ANOSIM) was used to evaluate the similarity of SCRS between within-group and between-group data at each time point (999 permutations; R version 3.0.3), with the results (R value) reflecting the degree of cellular response [18]. This provides a method to statistically test whether there is a significant difference between two or more groups of sampling units. ANOSIM analysis returns two important factors, the R value (ranging from − 1 to + 1), which is based on the ranks of dissimilarity between within-group and between-group data, with a greater value indicating greater dissimilarity, and the *p* value, which indicates whether the level of significance of any difference.

#### Random forest analysis

The Random Forest model was used to construct a classification model to analyse SCRS under different growth phases via default parameters (R package “randomForest”, ntree = 5000, using default mtry of sqrt(p), where p is the number of Raman bands) [18]. The model was evaluated internally with a tenfold cross validation [19]. For Random Forest discriminating cells among the lag phase, log phase, and stationary phase, about 20 of 30 cells from each triplicate of the three phases were selected randomly and combined for construction of a training dataset (n ≈ 70), and the rest of the cells were used to form a test dataset (n ≈ 30). The misclassification rates of both the training and test datasets were calculated to determine an optimal number of principal components (PCs). The test dataset was rotated into a new dataset of PCs by the loadings of the PCA of the training dataset, as described previously for converting two datasets in the same spectral space.

## Results and discussion

### Single-cell Raman spectroscopy for discrimination of different growth phases of *L. casei* Zhang

**Fig. 1:**
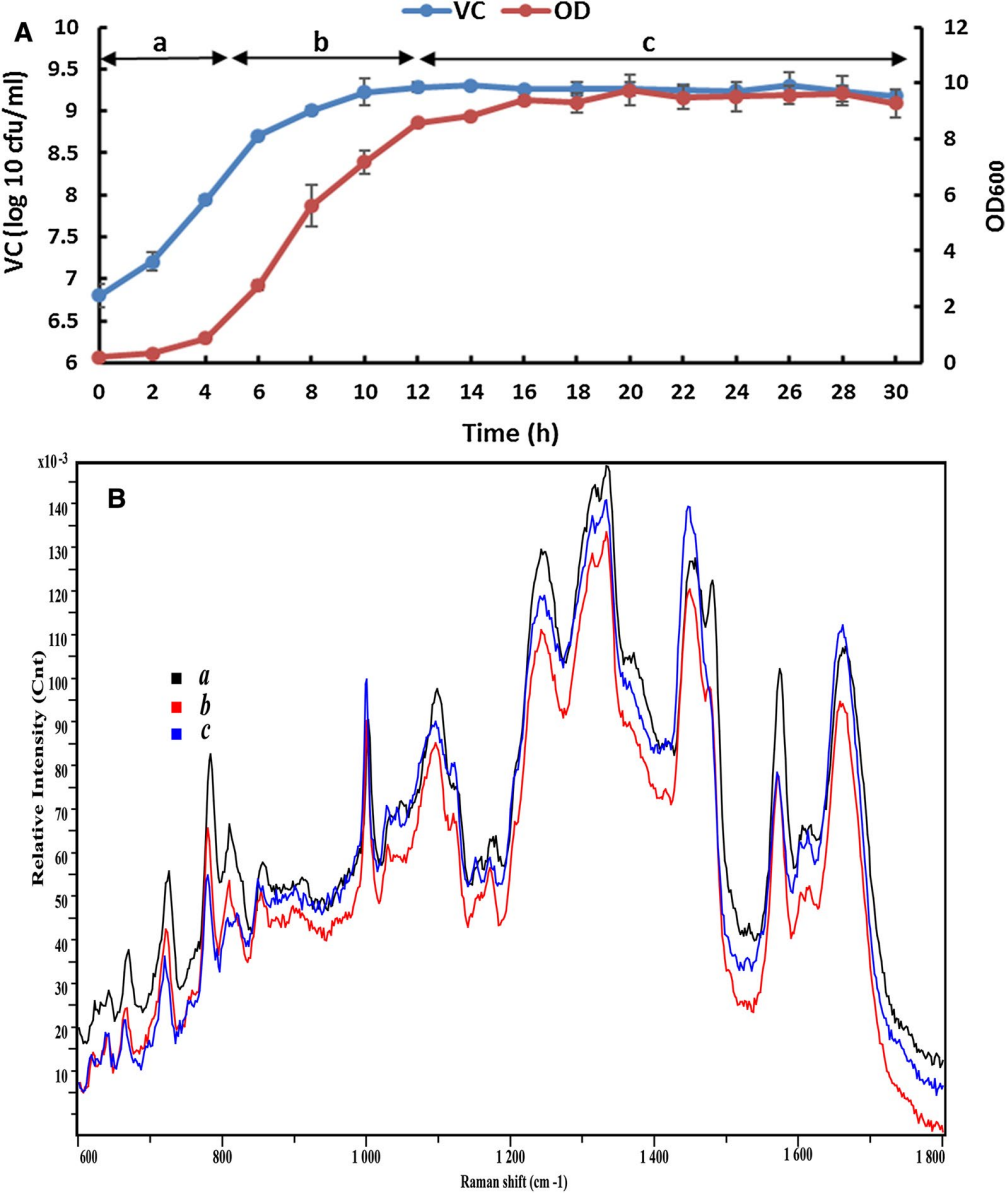
**A** A typical *L. casei* Zhang growth curve. **B** Mean Raman spectra of cells at three growth phases a–c. Raman spectra of *L. casei* Zhang cells taken at different growth phases on the growth curve. Cell samples for Raman analysis were obtained at the lag, log, and stationary growth phases, as indicated by the growth curve (portions a, b, and c, respectively). The three Raman spectra, representing different metabolic states, display significant changes in the Raman peak intensities and peak ratios

Figure 1A shows a normal *L. casei* Zhang growth curve displaying lag, log, and stationary growth phases. Cell samples for Raman analysis were obtained at different growth phases, as indicated by the growth curve (portions a, b, and c). Raman spectra of *L. casei* Zhang cells acquired during the (a) lag phase, (b) log phase, and (c) stationary phase are shown in Fig. 1B. Each spectrum is an average of 102 bacterial cells. The three Raman spectra, representing different metabolic states, display significant changes in Raman peak intensities and peak ratios.

**Fig. 2:**
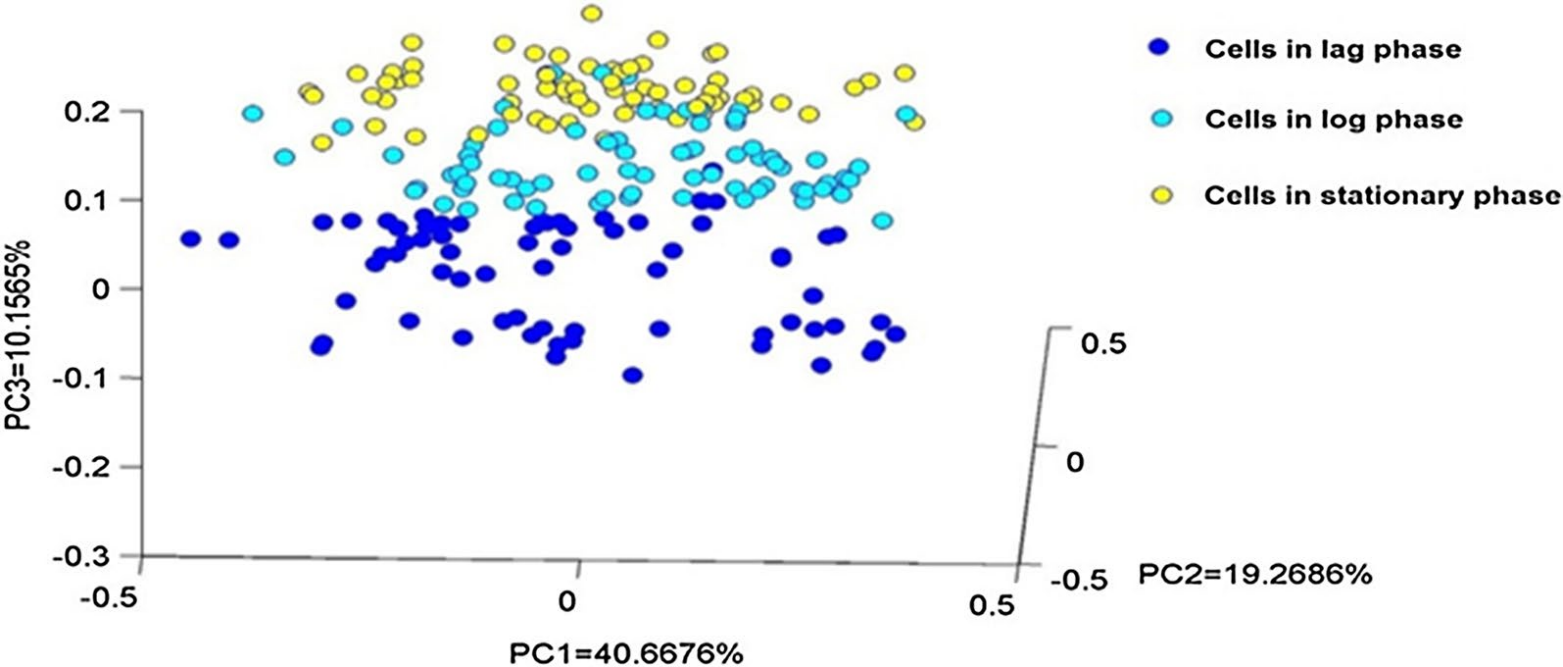
Principal component analysis (PCA) of Raman spectra for normal *L. casei* Zhang. PCA scatterplot using the PC1, PC2, and PC3 values. Raman spectra acquired at lag phase (1–4 h), log phase (8–14 h), and stationary phase (18–30 h) are represented by different symbols

Bacterial cells in the different metabolic states could be more clearly identified and visualised by performing PCA on the Raman spectra. The scatter plot of the first (PC1), second (PC2), and third (PC3) principal components (Fig. 2) shows three groups of cells that are clearly separated by their metabolic state. These three groups can be identified based on their time range on the growth curve (Fig. 1A): 1–4 h, corresponding to the lag phase (dark-blue dots); 8–14 h, corresponding to growth at the log phase (light-blue dots); and 18–30 h, corresponding to the stationary phase (yellow dots). Separation of the groups is better between the log phase (8–14 h) and the stationary phase (18–30 h) (Fig. 2). Bacterial cells at the log phase overlap slightly with bacterial cells at the stationary phase. The poor group separation for the latter stages of cell growth between the log and the stationary phases indicates that there is an increasingly heterogeneous population of cells at different growth stages, with some cells still actively undergoing metabolic changes, and others at rest [12].

**Fig. 3:**
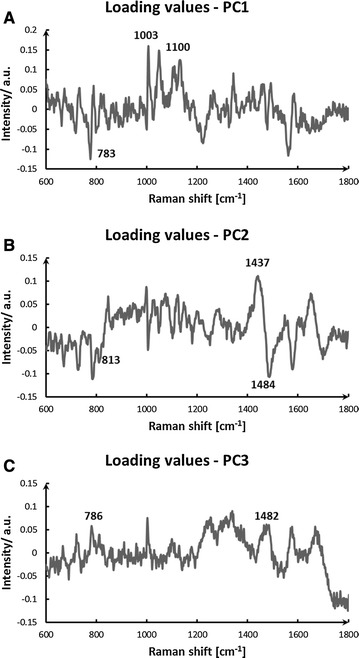
Different groupings of Raman spectra taken at different time points during normal cell growth. The loading values of PC1, PC2, and PC3 are plotted (parts **A**, **B**, and **C**, respectively). The peaks at 783, 1003, and 1100 cm^−1^ were identified for PC1, those at 813, 1437, and 1484 cm^−1^ were identified for PC2, and those at 786 and 1482 cm^−1^ were identified for PC3

**Fig. 4:**
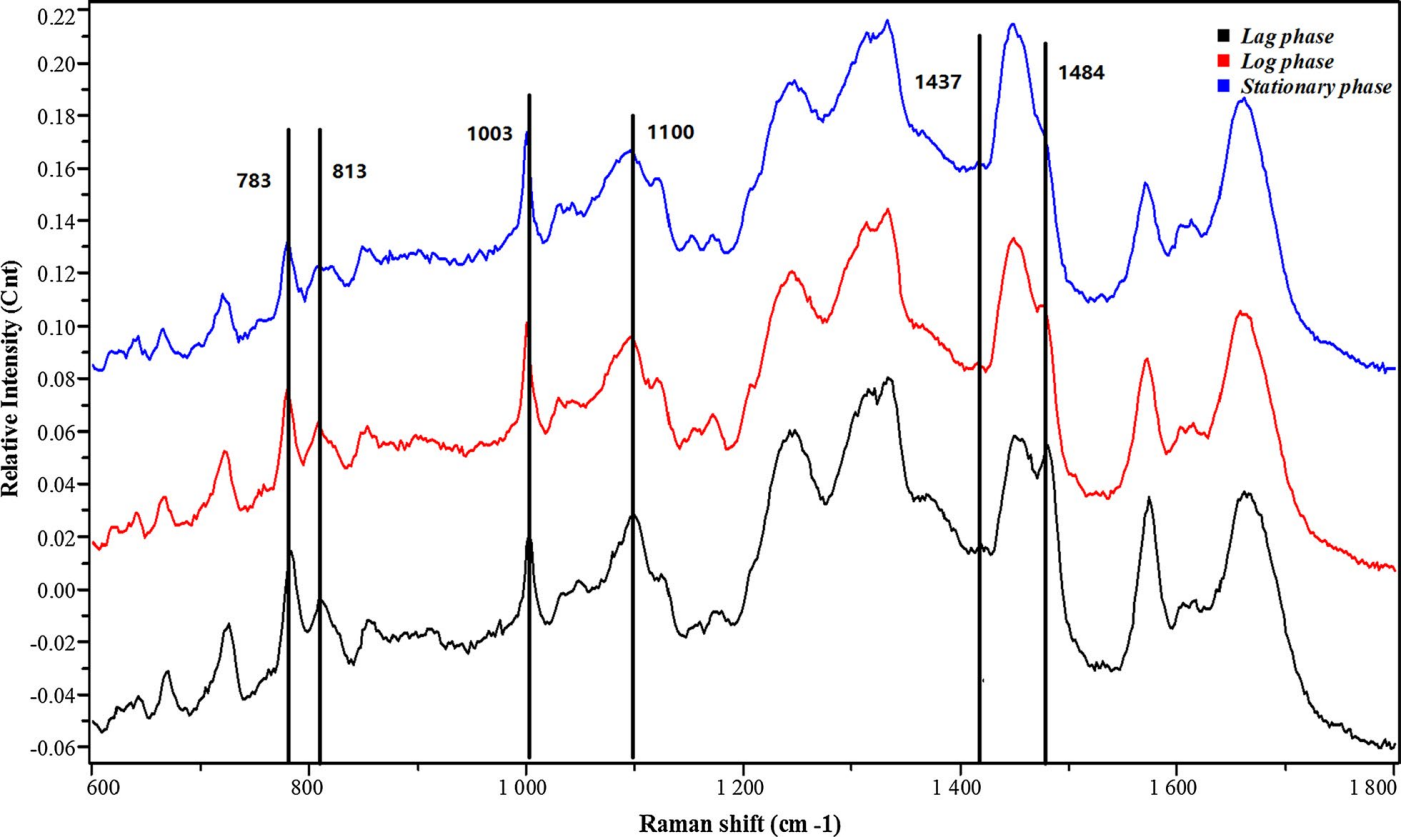
The most significant changes in spectral peaks during three growth phases of the bacterial. The primary Raman peaks that contribute to the data separation in the PCA plots from Fig. 3 are labelled

The loading values of PC1, PC2, and PC3 are plotted in Fig. 3 (parts a, b, and c, respectively). These indicate the Raman peaks that have the highest absolute variance over time and that contribute most to the separation observed in the PCA plot. PC1, PC2, and PC3 account for more than 70% of the variance in the data. The peaks of 783, 1003, and 1100 cm^−1^ were identified for PC1, those of 813, 1437, and 1484 cm^−1^ were identified for PC2, and those of 786 and 1482 cm^−1^ were identified for PC3. Figure 4 displays the most significant changes in spectral peaks during the bacterial growth. It also suggests that these peaks can be used as simple parameters to classify the metabolic state of an unknown *L. casei* Zhang bacterial cell following Raman analysis.

### Correlation of Raman spectroscopic changes to metabolic states of *L. casei* Zhang

Our SCRS study is a comprehensive analysis that provides more detailed spectral and temporal information for all Raman spectral components, including DNA/RNA-related, protein-related, and lipid-related peaks that change during cell growth. This detailed analysis is important for defining a benchmark for the variability in Raman spectra due to cell growth that can be used for identifying and predicting the growth phase of a single bacterial cell, as discussed in the next section.

**Fig. 5:**
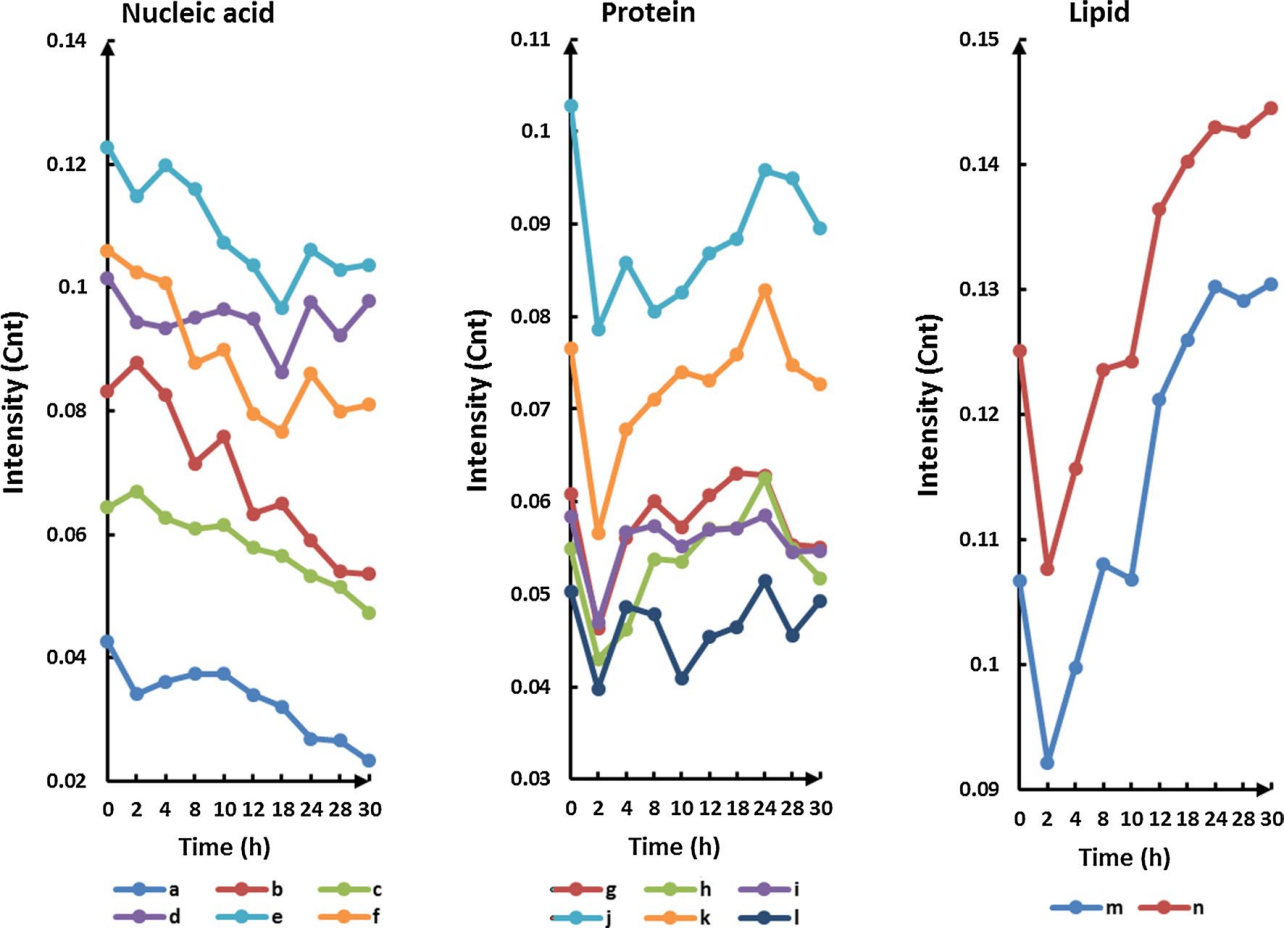
Time traces of relative Raman peak intensities during normal cell growth. Individual traces are labeled a throught. Peak traces were grouped based on similar profiles. Grouping of the Raman frequencies also coincided with the molecular species assigned to each of the peaks. The Raman frequencies in group A are specific to nucleic acids, those in group B are associated with protein, and those in group C are associated with lipids. a 668 cm^−1^; b 1575 cm^−1^; c 1100 cm^−1^; d 1484/1482 cm^−1^; e 783/786 cm^−1^; f 813 cm^−1^; g 934 cm^−1^; h 852 cm^−1^; i 963 cm^−1^; j 1032 cm^−1^; k 1550 cm^−1^; l 1003 cm^−1^; m 1437 cm^−1^; n 1443 cm^−1^

An analysis of the time-dependent changes in the individual Raman peaks is shown by the peak intensity time traces in Fig. 5. Three groups of Raman peaks can be identified based on similarities in their time-trace profiles. Moreover, the peaks also group according to the type of biomolecule to which they are assigned, as shown in Table 1 [20]. The Raman frequencies in group A are specific to nucleic acid, those in group B are associated with protein, and those in group C are associated with lipid. In general, most of the nucleic acid peaks exhibit a continuous decrease in intensity, whereas the protein and lipid peaks increase in intensity. However, several peaks exhibit a different trend. The 668- and 1575-cm^−1^ peaks in the nucleic acid group (labelled a and b) increase in intensity for the first 10 h but then decrease, and several protein peaks in the protein group show a minimal increase. The most significant changes in peak intensity can be observed for the 783 and 813-cm^−1^ Raman frequencies related to DNA/RNA. The peak intensities decrease by a factor of 2 during the transition from the log phase to the beginning of the stationary phase (points b to c in Fig. 1A), which can be associated with cell division [18]. Bacterial cells double the amount of DNA just before binary fission occurs, but have only one DNA complement during the stationary phase. The decrease in DNA/RNA-specific peak intensities and the increase in protein-specific and lipid-specific peak intensities over time indicate an increase in protein synthesis and lipid synthesis as a response to environmental stress induced by the depletion of nutrients. This is a known stress response for bacteria. The Raman spectra do not change significantly during the late stationary phase, as shown in Fig. 5 by the constant peak intensities after approximately 24 h. During the late stationary phase, no DNA or protein and lipid synthesis occurs because bacterial cell metabolism becomes inactive [12]. It is important to emphasise that even though no changes in the OD and viable count (VC) can be observed in the growth curve (Fig. 1A) after approximately 12 h, Raman spectroscopy is sensitive enough to detect changes in the bacterial cells up to 24 h. This is perhaps not surprising considering that traditional measurements (OD and VC) are only sensitive to the concentration of cells in the solution of the fermentation broth and not to physiological changes of cells themselves, whereas Raman spectroscopy can provide specific markers of DNA, RNA, protein, and lipid, whose ratios in a bacterial cell can be highly dependent on the cell’s metabolic state. These results highlight the importance of single-cell analysis over bulk-cell analysis for detecting and evaluating cell growth.

**Table 1:** Raman frequencies and their peak assignments grouped by their time-dependent trace profiles in Fig. 4

Curve	Raman bands (cm^−1^)	Biological assignment/interpretation
a	668	G ring breathing
b	1575	Guanine, adenine (ring stretching)
c	1100	Polyhydroxybutyrate
d	1484/1482	Amide II
e	783/786	Phosphodiester; cytosine
f	813	Nucleic acids (C–O–P–O–C in RNA backbone)
g	934	Proline, hydroxyproline, *v*(C–C) skeletal of collagen backbone
h	852	Tyr
i	963	CH_2_ rock, C–C_ɑ_
j	1032	Phe
k	1550	Tryptophan
l	1003	Phe
m	1437	CH_2_ and CH_3_ deformation vibrations (lipid)
n	1443	CH_2_ bending mode of proteins and lipids

Raman spectroscopy can provide specific markers of DNA, RNA, protein, and lipid, whose ratios in a bacterial cell can be highly dependent on the cell’s metabolic state

### Classification of single-cell Raman spectra to predefined different growth phases of *Lactobacillus casei* Zhang cells

**Fig. 6:**
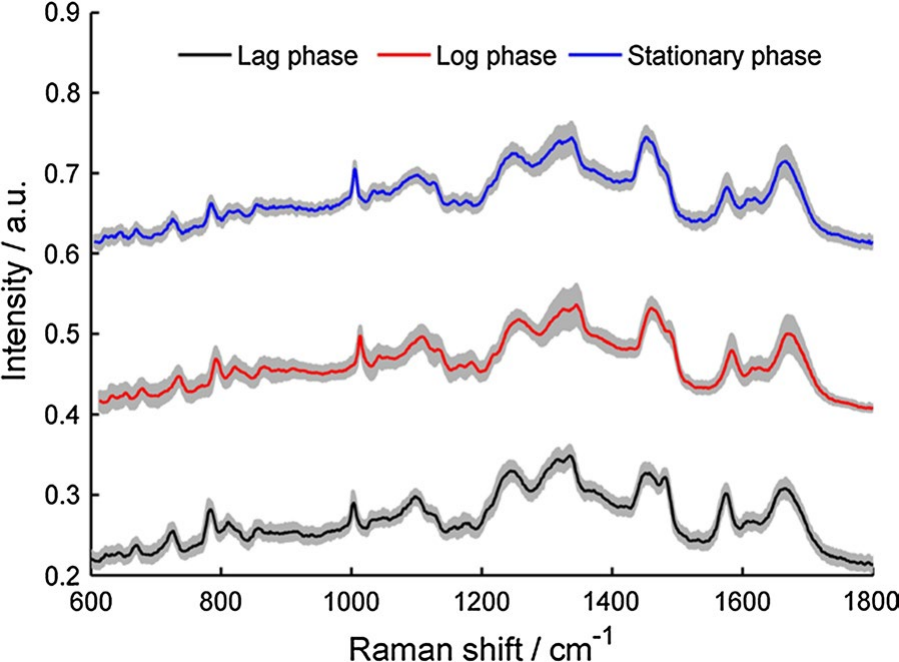
Mean Raman spectra and double standard deviation depicted as grey corona of all growth phases. A normalised double standard deviation was calculated for each type of cell. The mean of the standard deviation per channel was normalised to the standard deviation of the channel means, resulting in a standard deviation of the means (SDM), as showed in Table 2A

The aim at this stage in the process is to develop an automated algorithm that recognises and identifies cell types from different growth phases by RF. The major interest is the separation of log phase cells and stationary phase cells from all cells during LAB fermentation. Because probiotic and starter strains are generally harvested in the log or stationary growth phase for high cell densities in industrial production [21], a promising way to extract the miniscule intergroup differences is by interpreting the whole spectrum in a multivariate way as the spectral fingerprint. In-depth and exhaustive knowledge of each of the signals in the Raman signatures is not mandatory in these pattern-matching approaches; nonetheless, the whole information is considered in the data evaluation. However, an important premise for doing so is sufficient reproducibility of the spectra within the different growth phase cells, although the spectra from different time points are from different growth phases. After pre-processing, the treated spectra have to be analysed for inherent variations due to the fact that single cells are measured [18]; a significant distinction was detected at each of the growth states (*p* = 0.001 for each; ANOSIM) (Table 2A). Moreover, the distances between different growth states (as measured by the R value; ANOSIM) were 0.83 (lag, log), 0.71 (lag, stationary), and 0.89 (lag, stationary) (Table 2A), suggesting high dissimilarity of SCRS among the different growth phases. In addition, to compare the types of cells from different growth, a normalised double standard deviation was calculated for each type of cell [16]. The double standard deviation for each cell type is shown as a grey corona to the mean spectra displayed in Fig. 6. The mean of the standard deviation per channel is normalised to the standard deviation of the channel means, resulting in a standard deviation of the means (SDM) [16]. Thus, the standard deviation per channel is placed into a relation of a mean spectrum statistical property. The SDM values for each species are given in a rightmost column of Table 2A, where low SDM numbers represent low channel-wise variability and thus high reproducibility and reliability of the dataset. High numbers in the range of almost 1 would indicate high volatilities per channel and would cast doubt on the reliability of the data [18, 22]. All of the calculated SDMs are significantly lower than 1 and within a tight interval between 0.12 and 0.20. The lag phase cell spectrum (SDM 0.12) is the lowest, whereas log phase cells have the highest SDM (0.20), showing that the observed Raman information does not depend largely on a different batch. Consequently, the mean Raman data can be used to create a classification system.

**Table 2:** (A) Random Forest results for the classification model, (B) Identification of an independent test dataset from unknown samples

Identified as	True					
	Lag	Log	Stationary	Specificity (%)	SDM	ANOSIM
(A)						
Lag	67	4	0	97.1	0.12	0.83 (lag, log)**
Log	2	64	7	85.3	0.20	0.71 (log, stationary)**
Stationary	0	7	63	90	0.15	0.89 (lag, stationary)**
Sensitivity (%)	94.4	87.7	90	90.7		

Group	No. of correctly assigned cells	No. of total cells	Accuracy (%)	Total accuracy (%)
(B)				
Lag	25	26	96.2	
Log	31	36	86.1	91.2
Stationary	27	29	93.1	

The standard deviation of the means (SDM) of SCRS per sample was calculated (range from 0.12 to 0.20). Low SDM numbers represent high reproducibility and high reliability of the dataset. An analysis of similarity (ANOSIM) was performed to compare distances of between-group cells and within-group cells at different growth states. ** Represents *p* < 0.01. Lag, lag phase, log, log phase, stationary, stationary phase

The ability to predict exactly the type of cells at different metabolic stages during LAB fermentation would be an additional advantage of the vibrational spectroscopic technique. It is evident from Fig. 4 that the spectra of the different cell types cannot be distinguished by eye and that multivariate statistical methods are required for classification. Analogous to a previous report on dried cells [18], RF was used to develop a classification model. During the training processing, separating planes existed between the different classes. The model was validated internally by tenfold cross-validation. This means that the whole dataset of 214 spectra was split into 10 subsets; a classification model was trained with nine subsets, and the tenth subset to predict the accuracies was calculated. The resulting confusion table is shown in Table 2A. In total, 194 of 214 spectra were correctly classified, resulting in an accuracy of 90.7%. The best results were achieved for lag phase, for which 67 out of 71 single-cell spectra were classified correctly. This yields a mean sensitivity of 90.7% and a mean specificity of 90.8%. The specificities for the whole dataset range as 97.1% in the case of lag phase, 85.3% in the case of log phase, and 90% for stationary phase. Most of the misclassified log-phase spectra were classified as stationary phase (7 out of 73), and all misclassified stationary-phase spectra were classified as log phase (7 out of 70). This may be explained by an increasingly heterogeneous population of cells at log phase and stationary phase [12]. Fewer incorrect assignments occurred between lag phase and log phase (4 out of 71 lag phase spectra were falsely classified as log phase).

An independent group SCRS for use as a test group was obtained from different growth phases to confirm the predictive capacity of the classification model. The previously built RF model was used to predict this independent test dataset to check for overfitting. Table 2B summarises the results: 92.1% of spectra were assigned correctly (83 of 91 spectra); the accuracy ranged between 86.1% for log phase and 96.2% for stationary phase. These results suggest that the built RF model can be used to identify independent test data.

The use of an RF model showed that cell growth phase can be assessed with high accuracy, sensitivity, and specificity based on the Raman spectra of individual cells. This model served as a powerful database for the recognition and discrimination of the Raman spectra. A similar algorithm showed best results for classifying Raman spectra of different stress responses of *Escherichia coli* cells [18]. When employing such a model in the future, the steps entailing measurement of OD and VC could be omitted, and analysis of newly acquired data could be executed based on the previously established multivariate model. Then, Raman spectroscopy could facilitate non-invasive continuous monitoring of cell growth and metabolism in single cells, which is of particular interest in the field of LAB fermentation, such as for accurately determining the harvest time and activity of inoculum. However, to transform Raman spectroscopy into an online high-throughput assay, spectra acquisition times must be reduced. Shorter spectra acquisition times should not impact the specificity of multivariate classifications and could therefore be employed once classification models are established [23]. Automated cell culture monitoring systems could benefit from the implementation of Raman spectroscopy as a method for real-time monitoring of bacterial metabolism and growth states. Raman spectroscopy may offer the unique possibility of monitoring the early log, late log, and early stationary phases of cell growth without any marker in their native, unprocessed state [12]. Moreover, in future studies, to understand the metabolic pathway and activity during fermentation of LAB, we can consider combining SCRS and single-cell stable isotope (^13^C, ^15^N, and ^2^H) probing (SIP) to trace carbon, nitrogen, and hydrogen-related pathways or general metabolic activity. To date, DNA, RNA, and protein or lipid SIP have been developed for different metabolic pathways of different bacteria [15, 24–27].

## Conclusions

In this work, we demonstrated the use of SCRS for discriminating and predicting growth phases of individual bacterial cells of *L. casei* Zhang. The decrease in nucleic acid levels and the synthesis of lipids and proteins in three growth phases (lag, log, and stationary) could be tracked through time. To the best of our knowledge, this is the first reported method for predicting the growth phase of LAB cells at the level of individual live cells. Furthermore, we are able to monitor real-time changes of nucleic acid-related, protein-related, and lipid-related compounds at the single-cell level.

SCRS enables spectral analysis of individual live bacterial cells under near physiological conditions (MRS culture media) to develop a fundamental understanding of cell heterogeneities and their effects on microbial population dynamics. Additionally, SCRS is sensitive enough to detect changes in bacterial cells during the stationary phase. We have made relevant the use and potentialities of this approach for studying LAB and probiotics at both the industrial and the research level. Considering the great importance of the probiotics market, the systematic industrial implementation of these techniques should be explored much more than has occurred to present. In regard to future research, it is our opinion that using SCRS techniques to investigate LAB and probiotics represents a relatively novel approach that is far from being fully explored and for which much remains to be done.

## Abbreviations

OD: optical density
VC: viable count
LAB: lactic acid bacteria
RF: Random Forest
SCRS: single-cell Raman spectroscopy
*L.* casei Zhang: *Lactobacillus* casei Zhang
PCA: principal component analysis
ANOSIM: analysis of similarities
SIP: stable isotope probe
